# Identification of colorectal cancer immune biomarkers *via* eQTL mapping: a Mendelian randomization and transcriptomic analysis study

**DOI:** 10.7717/peerj.21070

**Published:** 2026-04-14

**Authors:** Genyuan Pu, Zi Yin, Xin Zhang, Zhiqi Liu, Caiting Yang, Chunping Xu, Mingming Lai

**Affiliations:** School of Basic Medicine, Dali University, Dali, China

**Keywords:** Colorectal cancer, Bioinformatics analysis, Molecular biomarkers, Immune cell infiltration, Targeted therapy and immunotherapy

## Abstract

**Background:**

Colorectal cancer (CRC) remains a leading cause of cancer-related mortality worldwide. The identification of effective molecular targets is crucial for advancing precision medicine and prognostic strategies. This study aims to uncover key CRC biomarkers through integrative bioinformatics analyses, providing mechanistic insights for therapeutic development.

**Methods:**

We analyzed three CRC datasets from the Gene Expression Omnibus (GEO) database. Expression quantitative trait loci (eQTL) analysis was performed to identify instrumental variables (IVs), which were subsequently used in Mendelian Randomization (MR) analysis with CRC Genome-Wide Association Study (GWAS) data. MR-associated genes were intersected with differentially expressed genes (DEGs) to screen disease-related key genes. Functional enrichment analyses were conducted using Gene Ontology (GO), Kyoto Encyclopedia of Genes and Genomes (KEGG), and Gene Set Enrichment Analysis (GSEA). Additionally, immune cell infiltration and gene-immune correlation analyses were performed. Finally, validation was performed using independent GEO, The Cancer Genome Atlas (TCGA) datasets, summary data-based Mendelian randomization (SMR) and quantitative reverse transcription polymerase chain reaction (qRT-PCR) in CRC cell lines.

**Results:**

A total of 776 upregulated and 981 downregulated DEGs were identified. Nine key genes were prioritized: *ATP13A4, CD1C, METTL7A, SLC18A1, CREB5, CXCR1, GZMB, HECW2,* and *TEAD2*, predominantly involved in cytokine receptor interaction pathways. CIBERSORT analysis revealed increased activated CD4+ memory T cells and M0 macrophages, alongside decreased plasma cells and natural killer (NK) cells in CRC. Key genes demonstrated significant correlation with immune cell subsets (*e.g.*, neutrophils, mast cells), highlighting their role in CRC immunobiology. Validation *via* SMR and qRT-PCR assays demonstrated significant dysregulation of four target genes (*CXCR1*, *HECW2*, *ATP13A4*) (*P* < 0.05).

**Discussion:**

This study suggests that *CXCR1*,* HECW2*, and *ATP13A4* may be involved in CRC development, providing a reference for targeted and immunotherapy research.

## Introduction

Colorectal cancer (CRC) is a prevalent malignant tumor of the gastrointestinal tract and constitutes a leading cause of cancer-related mortality worldwide. It ranks as the second most common cancer in women and the third in men globally (Bray et al., 2024; Kumar et al., 2021; Siegel et al., 2023). Although advancements in screening and treatment have contributed to declining mortality rates of CRC in some countries, the incidence continues to rise in many regions, exacerbating the associated medical burden (Clark et al., 2023; Dekker et al., 2019; Santucci et al., 2024). Despite significant progress in CRC research, identifying sensitive and effective therapeutic targets remains a critical challenge. The pronounced intratumor heterogeneity inherent to CRC facilitates metastasis and recurrence, major contributors to its high mortality rate (Zheng et al., 2020). Consequently, achieving precision therapy for CRC persists as a significant challenge, underscoring the urgent need to identify effective diagnostic, therapeutic, and prognostic targets for this disease.

Expression quantitative trait loci (eQTL) map associations between specific single nucleotide polymorphisms (SNPs) and quantitative variations in individual gene expression levels (Zhu et al., 2016). By analyzing the relationship between genomic variation and gene expression intensity across large samples, eQTL analysis elucidates the potential molecular-level impact of genetic variations on disease pathogenesis (Jansen et al., 2017). This approach is pivotal for deciphering the molecular mechanisms underlying diseases and identifying potential drug targets.

Mendelian randomization (MR) leverages genetic variants as instrumental variables to infer causal relationships between exposures and disease outcomes, offering a statistically robust approach to overcome these limitations (Sekula et al., 2016). MR provides insights into how modifiable exposures influence disease risk, driving its growing application in biomedical research (Richmond & Davey Smith, 2022). In particular, summary data-based Mendelian randomization (SMR), which integrates cis-eQTL and GWAS summary statistics, enables the prioritization of genes whose expression changes are potentially causally linked to disease risk. Although MR has been increasingly applied in cancer research, SMR-based investigations focusing on CRC-related gene expression regulation remain limited, and the functional relevance of many genetically prioritized genes has not been systematically explored within the tumor immune microenvironment.

**Figure 1:**
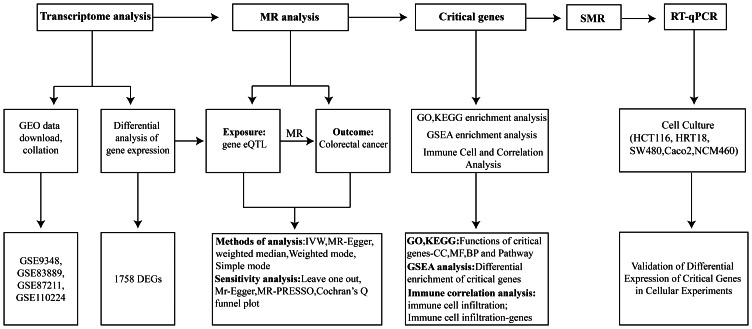
Workflow diagram of this study.

In this study, we applied an integrative analytical framework combining SMR analysis with transcriptomic validation to identify genes with putative causal effects on CRC risk. To further characterize the biological relevance of these genes, we assessed their expression patterns in CRC tissues, evaluated their associations with immune cell infiltration using the CIBERSORT algorithm, and explored their underlying molecular mechanisms through Gene Set Enrichment Analysis (GSEA). By integrating genetic causality inference with tumor immune context and functional pathway analysis, this study aims to refine the identification of CRC-associated genes with higher biological plausibility and translational potential. The overall workflow of this study is illustrated in Fig. 1.

## Materials and Methods

### Data collection and preprocessing

Gene expression microarray datasets and corresponding clinical phenotype data for CRC were retrieved from the Gene Expression Omnibus (GEO) database (https://www.ncbi.nlm.nih.gov/geo/). A total of four datasets were included in this study: GSE9348 (12 normal samples and 70 CRC samples), GSE83889 (35 normal samples and 101 CRC samples), GSE87211 (160 normal samples and 203 CRC samples), and GSE110224 (17 normal samples and 17 CRC samples). The GSE9348, GSE83889, and GSE87211 datasets were merged into a combined dataset (comprising 207 normal samples and 374 CRC samples), while GSE110224 served as the validation dataset (Table 1). Additionally, mRNA expression data for CRC (TCGA-CRC.htseq_fpkm) were obtained from The Cancer Genome Atlas (TCGA) database (https://www.cancer.gov/ccg/research/genome-sequencing/tcga) as an external independent validation cohort.

**Table 1:** Summary of microarray datasets utilized in this study.

GSE ID	Platform	Colorectal cancer	Normal	Date type
GSE9348	GPL570	70	12	Training
GSE83889	GPL10558	101	35	Training
GSE87211	GPL13497	203	160	Training
GSE110224	GPL570	17	17	Testing

### Differential Expression Genes (DEGs) analysis

The “sva” and “limma” R packages were employed to read and preprocess the GSE9348, GSE83889, and GSE87211 datasets (Chen et al., 2011; Ritchie et al., 2015). First, genes common to all datasets were retained to ensure data consistency, and batch effects were eliminated using the ComBat algorithm implemented in the “sva” package (Varma, 2020). Principal Component Analysis (PCA) was subsequently used to visualize and validate the efficacy of batch effect correction (*i.e.,* to compare sample clustering patterns before and after correction). The preprocessed datasets were then merged into a combined dataset. DEGs analysis was performed using the “limma” package to compare 207 normal samples and 374 CRC samples. The significance thresholds for identifying DEGs were set as adjusted *P*-value < 0.05 and —Fold Change— > 1.5 (Xia et al., 2022). DEGs were visualized using heatmaps generated by the “pheatmap” package and volcano plots created with the “ggplot2” package (Ito & Murphy, 2013).

### MR method

#### Data sources for MR

MR analysis was employed using genome-wide association study (GWAS) data to investigate potential causal relationships between genes and CRC. The GWAS data for the exposure was sourced from the eQTL dataset in the OpenGWAS database (https://gwas.mrcieu.ac.uk/), encompassing a total of 19,942 genes. The GWAS data for the outcome was obtained from the UK Biobank database (http://www.nealelab.is/uk-biobank), which included a sample size of 377,673. Both exposure and outcome GWAS data were restricted to individuals of European ancestry. The GWAS summary data used in this study are publicly available, and no additional ethical approval or informed consent was required.

### Instrumental variable selection (IVs)

Instrumental variables (IVs) were selected as follows: (1) SNPs significantly associated (*P* < 5 ×10^−^^8^) with gene expression levels were identified using the “TwoSampleMR” package (Hemani et al., 2018). (2) A linkage disequilibrium condition (R^2^ < 0.001, physical distance threshold of 10,000 base pairs) was applied to identify strongly correlated SNPs, retaining those with the most significant association to gene expression for subsequent analysis. (3) The instrument strength for each exposure was evaluated using the F-statistic, with IVs exhibiting F < 10, indicating potential weak instrument bias, being excluded from subsequent analysis (Burgess & Thompson, 2011).

### MR analysis

A two-sample MR analysis was conducted using the “TwoSampleMR” and “VariantAnnotation” packages (Sanderson et al., 2022). Causal effects were primarily estimated using the inverse variance-weighted (IVW) method. Supplementary analysis employed the MR-Egger, weighted median, weighted mode, and simple mode methods (Kong et al., 2024). Genes were considered potentially causally associated with CRC if they met the following criteria (Ference, Holmes & Smith, 2021): (1) IVW *P* < 0.05. (2) Consistent effect directionality across MR methods. (3) Exclusion of pleiotropic genes with *P* < 0.05. Heterogeneity among IVs was assessed using Cochran’s Q test (*P* > 0.05 indicating no significant heterogeneity) (Tian, Tom & Burgess, 2024). Horizontal pleiotropy was evaluated using the MR-Egger intercept test (Bowden et al., 2016). Leave-one-out analysis was performed to assess the influence of individual SNPs on the overall causal estimate (Liang et al., 2023). Multiple testing correction was applied using the false discovery rate (FDR) method.

### Identification and validation of key CRC genes

Genes identified by MR analysis were categorized as low-risk (Odds Ratio, OR < 1) or high-risk (OR > 1). Venn diagrams identified overlapping genes between these risk categories and the DEGs (upregulated and downregulated). Forest plots visualized ORs and confidence intervals from IVW and weighted median analyses for strongly correlated genes. Expression levels of key candidate genes were validated using the GSE110224 dataset. The genomic circles of genes and chromosomes were created using the “circlize” package (Gu et al., 2014).

### GO/KEGG enrichment analysis

GO functional annotation and KEGG enrichment analysis of the key genes were performed using the “ClusterProfiler” R package. The analysis results were filtered based on strict criteria: statistical significance was defined as *P* < 0.05, and the false discovery rate (FDR) was set at < 0.05 (Yu et al., 2012).

### GSEA

GSEA was conducted using the “ClusterProfiler” package and the “c2.cp.kegg.v2023.2.Hs.symbols” gene set collection from the MSigDB database (https://www.gsea-msigdb.org/gsea/) (Liberzon et al., 2011). Gene sets with a nominal *P*-value < 0.05 were considered significantly enriched.

### Immune cell infiltration and correlation analysis

Based on the “LM22” file, the “CIBERSORT” package was used to analyze the infiltration levels of immune cells in the control and experimental groups (*P* < 0.05), and box plots and bar charts were employed to observe the differences in immune cell levels (Yang et al., 2021). Finally, the “linkET” and “ggplot2” packages were used to visualize the associations between the infiltrating immune cell types in the disease group and the key genes related to the disease (Ito & Murphy, 2013).

### SMR analysis

SMR analysis was performed to further assess whether the associations identified by two-sample MR were driven by shared causal variants rather than linkage disequilibrium. cis-eQTL summary statistics were obtained from the OpenGWAS database, and CRC GWAS summary data were derived from the UK Biobank.

Only cis-eQTLs located within ±1 Mb of the transcription start site of each gene were considered, and the top associated cis-eQTL was selected as the instrumental variable. The heterogeneity in dependent instruments (HEIDI) test was applied to exclude associations driven by linkage disequilibrium, with a HEIDI *P* value > 0.05 indicating no significant heterogeneity. SMR analyses were conducted using the SMR software (version 1.3.1).

### Cell culture

Human CRC cell lines (HCT116, HRT18, Caco2, and SW480) and the normal human colorectal epithelial cell line NCM460 were obtained from Procell Life Science & Technology Co., Ltd. (Wuhan, China). NCM460 cells were cultured in RPMI-1640 medium supplemented with 10% fetal bovine serum (FBS); HCT116 and HRT18 cells were maintained in their recommended media with 10% FBS; Caco2 cells were cultured in DMEM supplemented with 20% FBS and 1% non-essential amino acids; and SW480 cells were cultured in DMEM supplemented with 10% FBS. All cell lines were incubated at 37 °C in a humidified atmosphere containing 5% CO_2_.

### RNA extraction

Total RNA was extracted from cell monolayers (≥90% confluence in 6-well plates) using TRNzol Universal Reagent (Tiangen Biotech, China). Cells were washed with PBS, lysed on ice in 500 µL TRNzol Universal for 5 min, and processed according to the manufacturer’s protocol (including chloroform separation, isopropanol precipitation, and 75% ethanol washes). RNA pellets were dissolved in DEPC-treated water. RNA concentration and purity (A_2__6__0_/A_2__8__0_ ratio) were quantified using a NanoDrop One spectrophotometer (Thermo Fisher Scientific, USA).

### Reverse transcription quantitative PCR (RT-qPCR)

Complementary DNA (cDNA) was synthesized from 1 µg of total RNA using HiScript III RT SuperMix (Vazyme, China) under the following conditions: 50 °C for 15 min, 85 °C for 5 s. Quantitative PCR was performed using HS Universal qPCR Master Mix and ROX plus (ACE Biotechnology, China) with the following cycling parameters: initial denaturation at 95 °C for 1 min 30 s; 40 cycles of 95 °C for 5 s and 60 °C for 30 s. Reactions were performed in triplicate. GAPDH served as the endogenous control. Relative gene expression was calculated using the 2ˆ(−ΔΔCt) method. The primers were synthesized by Sangon Biotech (Shanghai, China and the sequences were provided in Table S1.

### Statistical analysis

Data analysis was performed using SPSS 20.0. Graphical representations were generated using GraphPad Prism 8.0. Differences between two groups were assessed using an independent samples Student’s *t*-test. Statistical significance was defined as *P* < 0.05.

## Results

### Differential gene expression analysis

**Figure 2:**
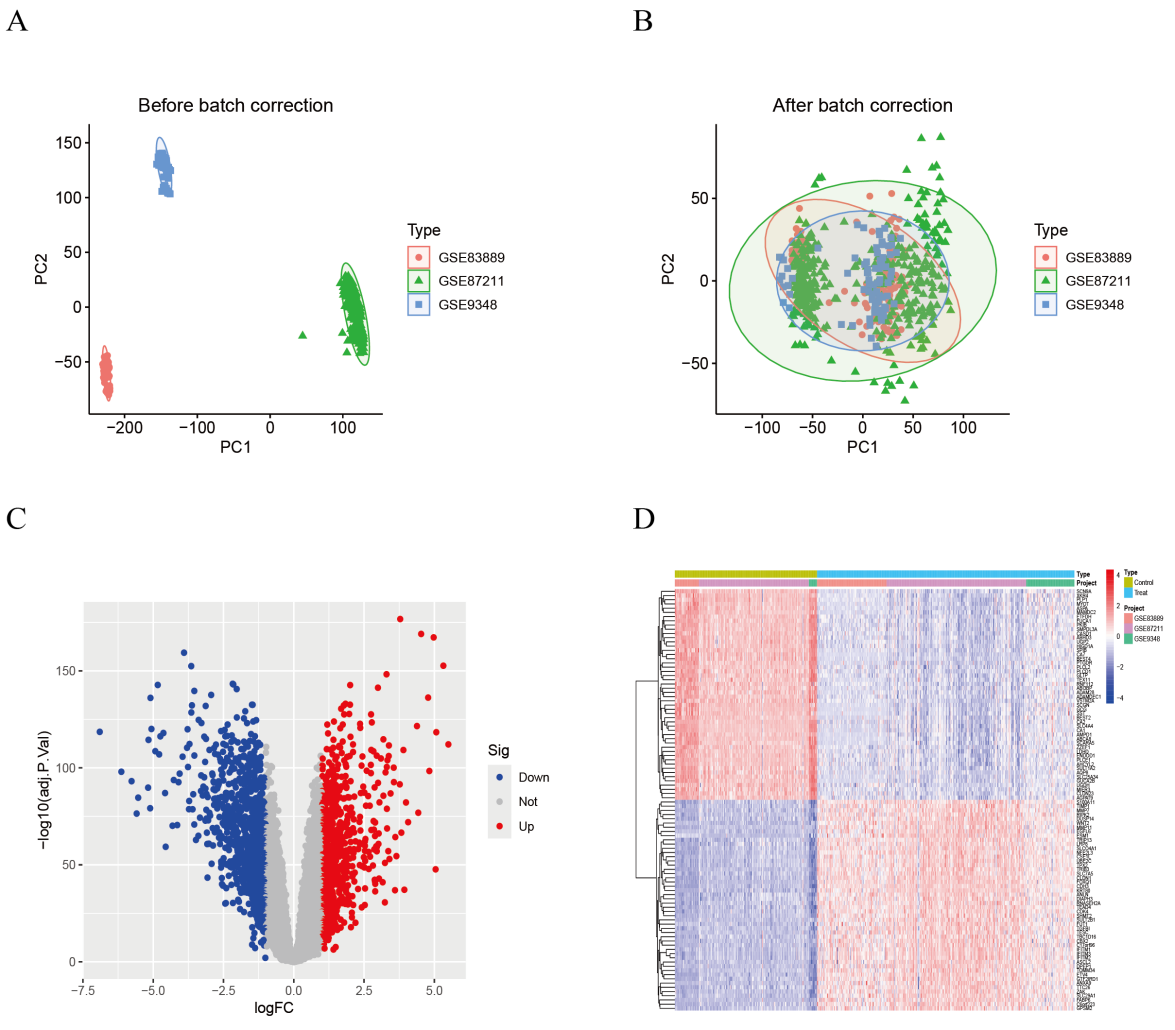
Batch effect correction and differential expression analysis. (A) PCA plot before batch correction. (B) PCA plot after batch correction. (C) Volcano plot of differentially expressed genes. (D) Heatmap of differentially expressed genes.

Following batch effect correction (Figs. 2A–2B), the integrated dataset comprised 207 normal samples and 374 CRC samples (Table 1). Differential gene expression analysis was performed on the CRC training cohort and identified a total of 1,758 differentially expressed genes (DEGs), consisting of 777 upregulated genes and 981 downregulated genes (Table S2). These findings are visualized in the corresponding volcano plot and heatmap (Figs. 2C–2D).

### IVs and MR analysis

After removing SNPs in linkage disequilibrium and excluding those with F-statistic <10, 26,152 SNPs exhibiting strong associations with genes were retained as valid instrumental variables (IVs) (Table S3). Two-sample MR analysis was subsequently conducted to assess the causal effect of each SNP on CRC risk. Sensitivity analyses were demonstrated no significant pleiotropy (MR-Egger intercept *P* > 0.05), and a significant association was observed using the IVW method (*P* < 0.05). These processes identified 221 disease-associated genes (Table S4).

**Figure 3:**
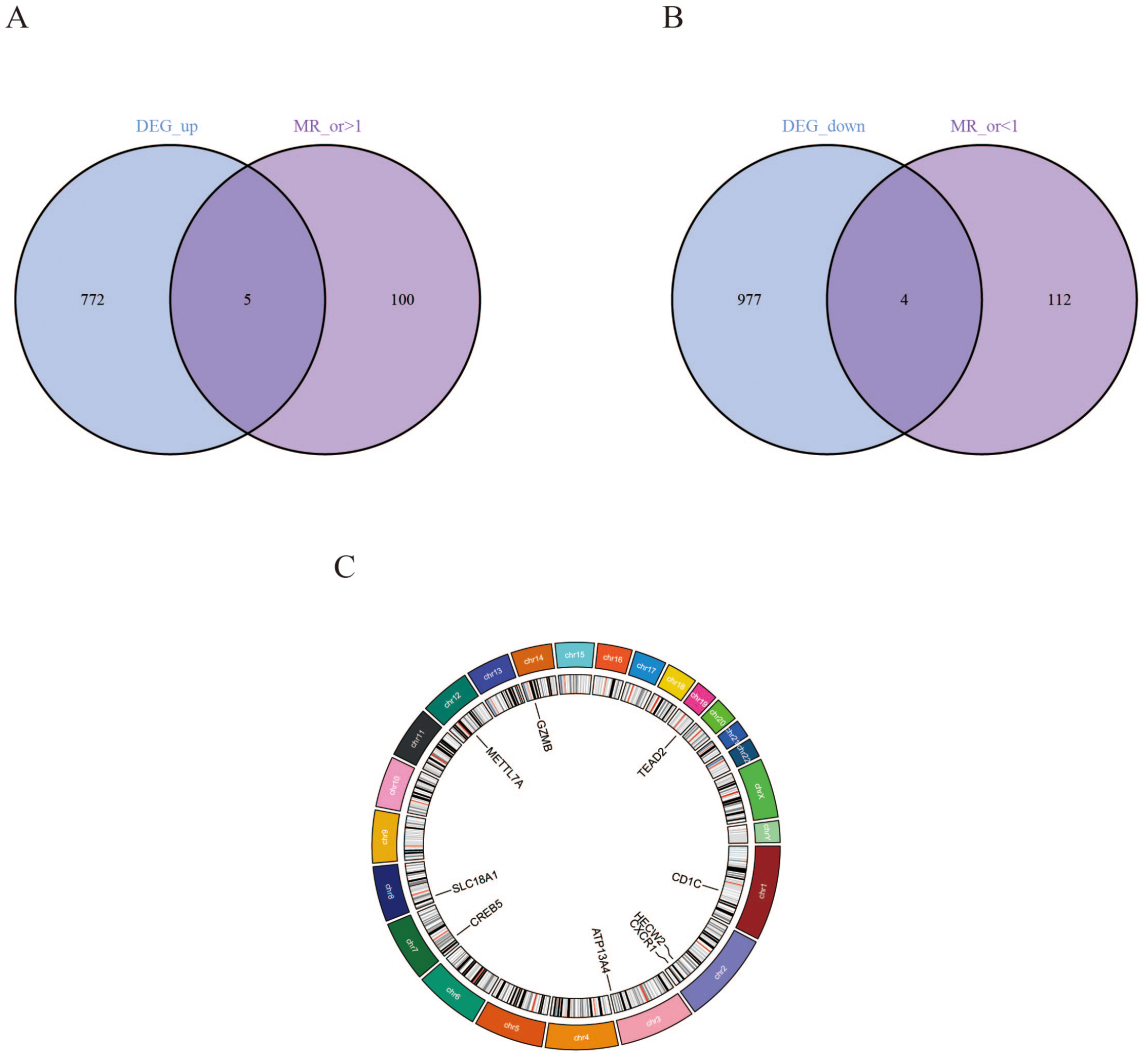
Screening and genomic localization of critical CRC-associated genes. (A) Intersection of disease-upregulated DEGs with MR-derived genes exhibiting OR > 1. (B) Intersection of disease-downregulated DEGs with MR-derived genes exhibiting OR < 1. (C) Chromosomal positions of the identified disease-critical gene.

**Figure 4:**
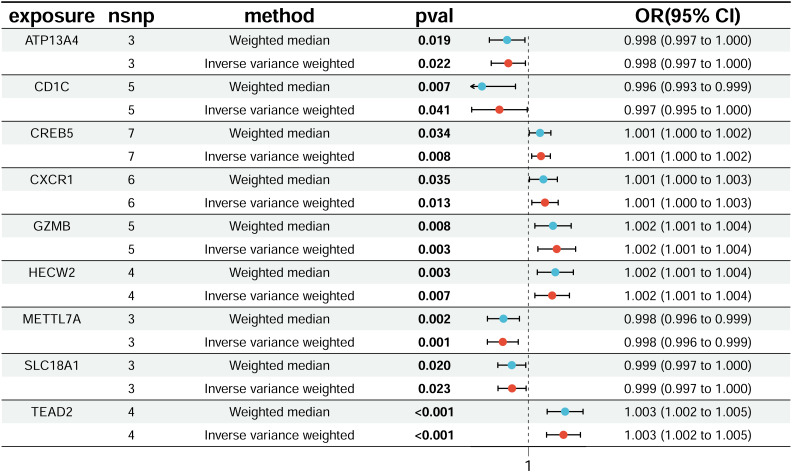
Disease-critical genes demonstrating causal associations with CRC risk.

To refine the risk assessment of these genes, we intersected the upregulated DEGs with MR-derived high-risk genes, yielding five genes associated with high CRC risk. Similarly, intersecting the downregulated DEGs with MR-derived low-risk genes identified four genes associated with low CRC risk. In total, nine disease-related co-expressed genes were identified, and their chromosomal locations were annotated (Figs. 3A–3C). Specifically, *CREB5*, *CXCR1*, *GZMB*, *HECW2*, and *TEAD2* were classified as high-risk genes, while *ATP13A4*, *CD1C*, *METTL7A*, and *SLC18A1* were identified as low-risk genes (Fig. 4). The results after FDR correction are detailed in Table S7.

To establish the causal relationship between these nine key genes and CRC, MR analysis was performed. The results demonstrated significant positive causal effects for the upregulated genes: *CREB5* (OR = 1.001; 95% confidence interval (CI) [1.000–1.002]; *P* = 0.008), *CXCR1* (OR = 1.001; 95% CI [1.000–1.003]; *P* = 0.013), *GZMB* (OR = 1.002; 95% CI [1.001–1.004]; *P* = 0.003), *HECW2* (OR = 1.002; 95% CI [1.001–1.004]; *P* = 0.007), and *TEAD2* (OR = 1.003; 95% CI [1.002–1.005]; *P* < 0.001). Conversely, the downregulated genes exhibited significant negative causal effects: *ATP13A4* (OR = 0.998; 95% CI [0.997–1.000]; *P* = 0.022), *CD1C* (OR = 0.997; 95% CI [0.995–1.000]; *P* = 0.041), *METTL7A* (OR = 0.998; 95% CI [0.996–0.999]; *P* = 0.001), and *SLC18A1* (OR = 0.999; 95% CI [0.997–1.000]; *P* = 0.023).

### Sensitivity analysis and validation of key genes

**Figure 5:**
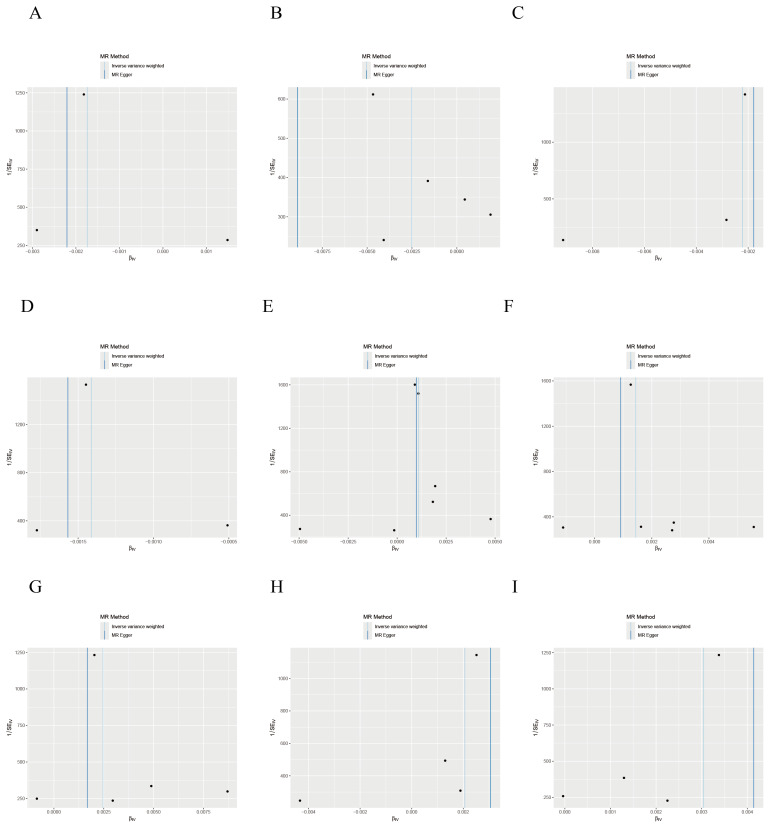
Funnel plot of MR analysis for the association between disease-critical genes and CRC risk. (A–I) *ATP13A4, CD1C, METTL7A, SLC18A1, CREB5, CXCR1, GZMB, HECW2, TEAD2*.

**Figure 6:**
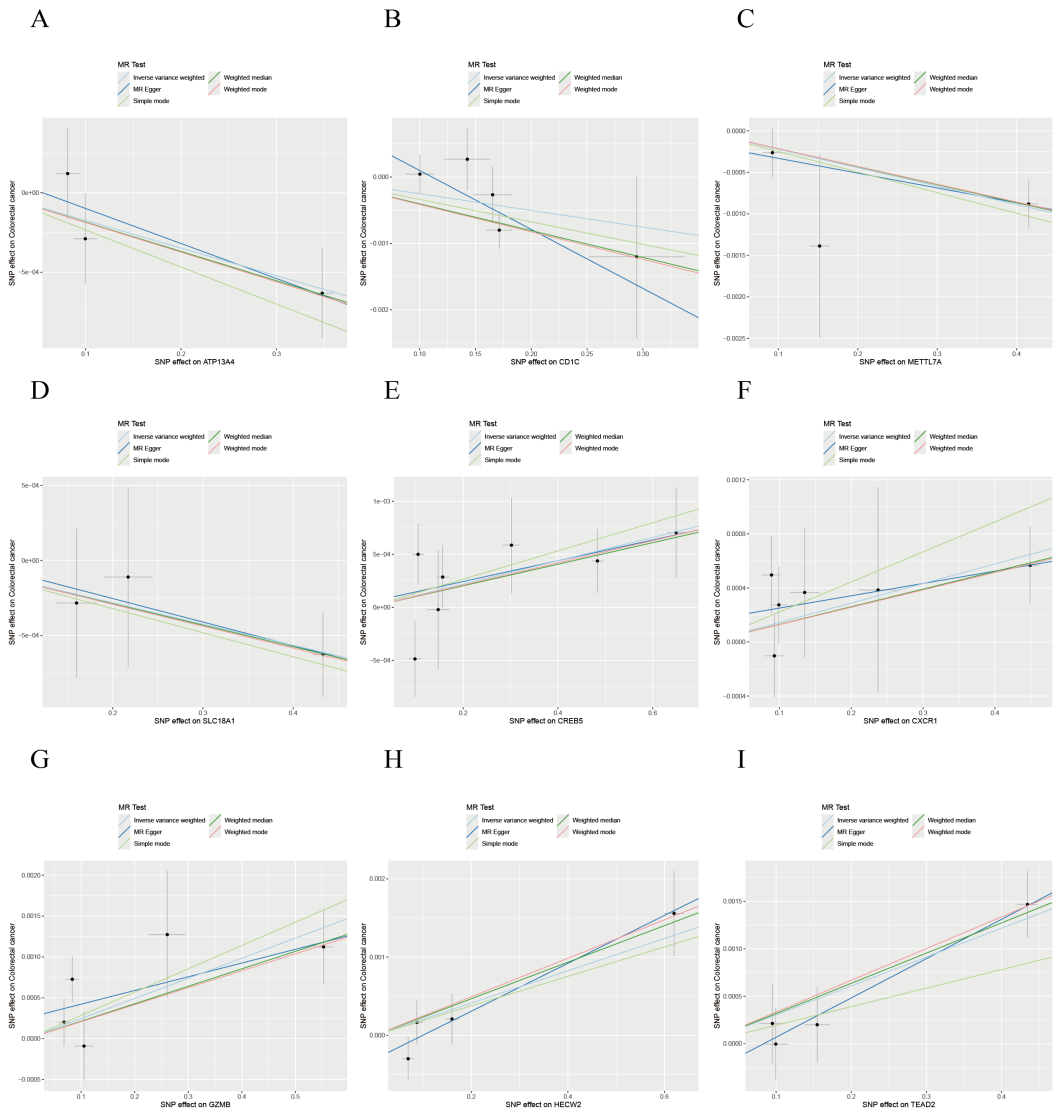
Scatterplot of MR analysis for the association between disease-critical genes and CRC risk. (A–I) *ATP13A4, CD1C, METTL7A, SLC18A1, CREB5, CXCR1, GZMB, HECW2, TEAD2*.

Sensitivity analyses, including MR-Egger regression and Cochran’s Q test, confirmed the absence of significant directional pleiotropy (MR-Egger intercept *P* > 0.05) or heterogeneity (Cochran’s Q *P* > 0.05) for the nine key genes, supporting the robustness of the MR findings (Table 2). Funnel plot analysis indicated that no single SNP disproportionately influenced the results or violated MR assumptions (Fig. 5). Leave-one-out analysis further corroborated the absence of horizontal pleiotropy, reinforcing the causal inferences (Fig. 6, Fig. S1). Additionally, external validation using the GSE110224 dataset and the TCGA database confirmed significant differential expression of the key genes in CRC tissues, consistent with our prior results (Fig. S2).

**Table 2:** Sensitivity analysis of disease-critical genes.

**Gene**	**P**_*MR*−*EGGER*_	**P**_*MR*−*Egger*.*Q*_	**P**_**IVW.Q**_
ATP13A4	0.348	0.359	0.600
CD1C	0.142	0.442	0.304
CREB5	0.227	0.396	0.517
CXCR1	0.382	0.711	0.750
GZMB	0.240	0.279	0.276
HECW2	0.102	0.586	0.406
METTL7A	0.395	0.372	0.619
SLC18A1	0.558	0.733	0.940
TEAD2	0.087	0.884	0.732

### Functional enrichment analysis of disease-critical genes

**Figure 7:**
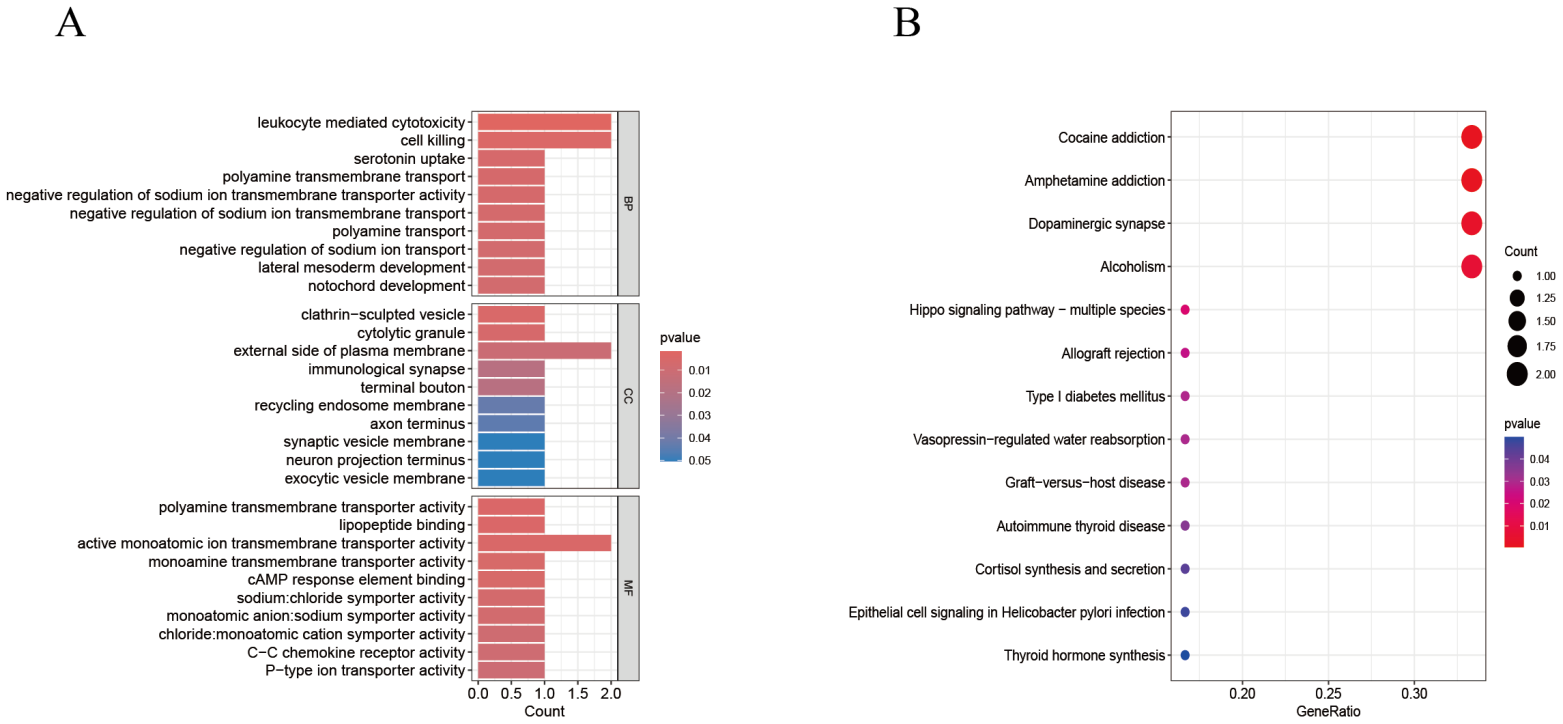
GO/KEGG. (A) GO enrichment analysis of CRC critical genes. (B) KEGG enrichment analysis of CRC critical genes.

To explore the potential roles of the nine genes, we performed GO and KEGG analyses. GO analysis revealed significant enrichment of the key genes in specific biological processes (BP), cellular components (CC), and molecular functions (MF). The results revealed that the key genes were primarily enriched in leukocyte-mediated cytotoxicity and cell killing (BP), the external side of the plasma membrane (CC), and the activity of active monovalent ion transmembrane transporters (MF) (Table S5). Additionally, KEGG pathway analysis indicated that the key genes were significantly enriched in pathways related to cocaine addiction, amphetamine addiction, and other associated pathways (Figs. 7A–7B, Table S6).

### GSEA of key genes

**Figure 8:**
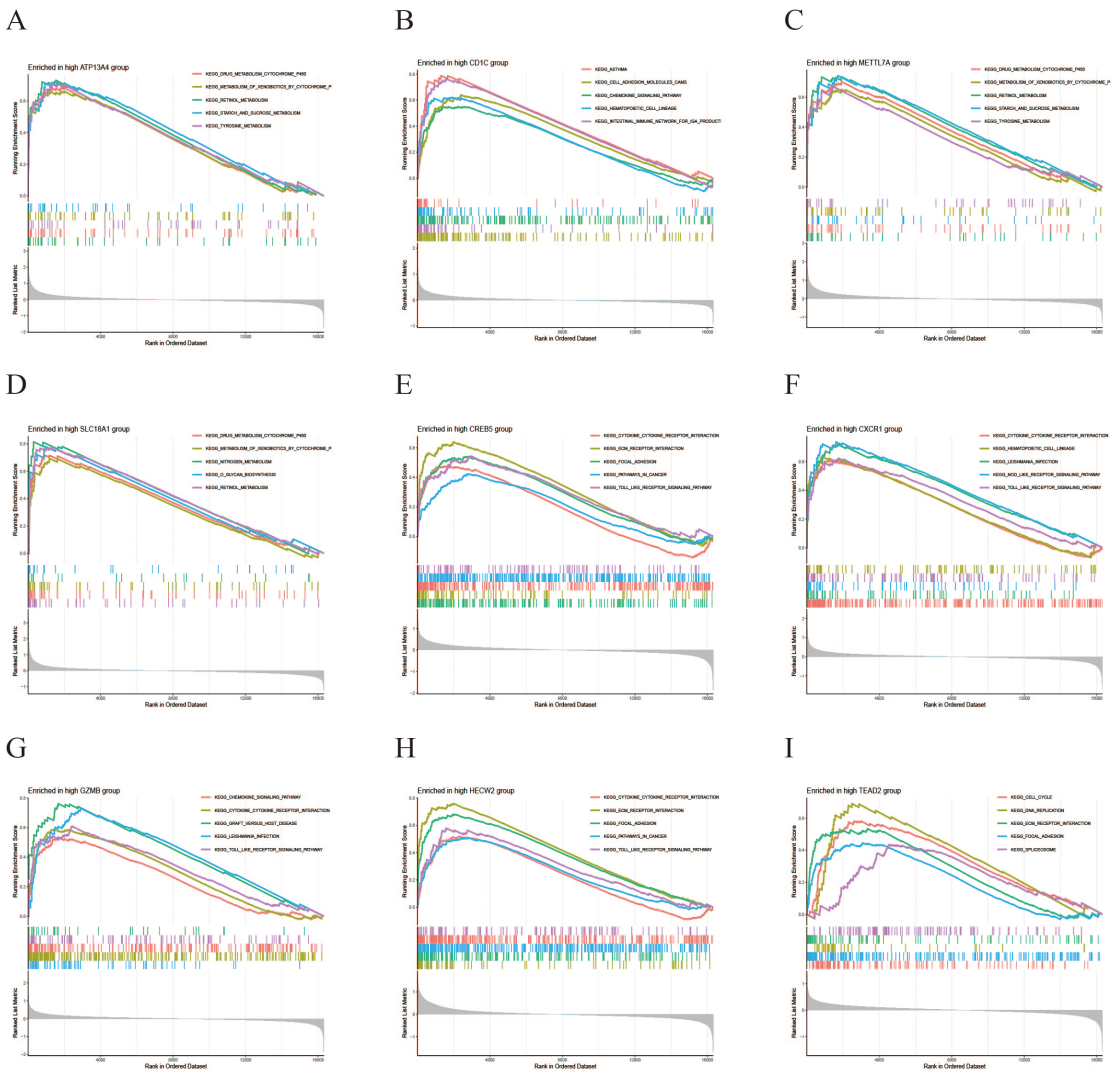
GSEA for high expression levels of disease-critical genes in CRC. (A–I) GSEA enrichment results for high expression of *ATP13A4, CD1C, METTL7A, SLC18A1, CREB5, CXCR1, GZMB, HECW2, TEAD2*.

**Figure 9:**
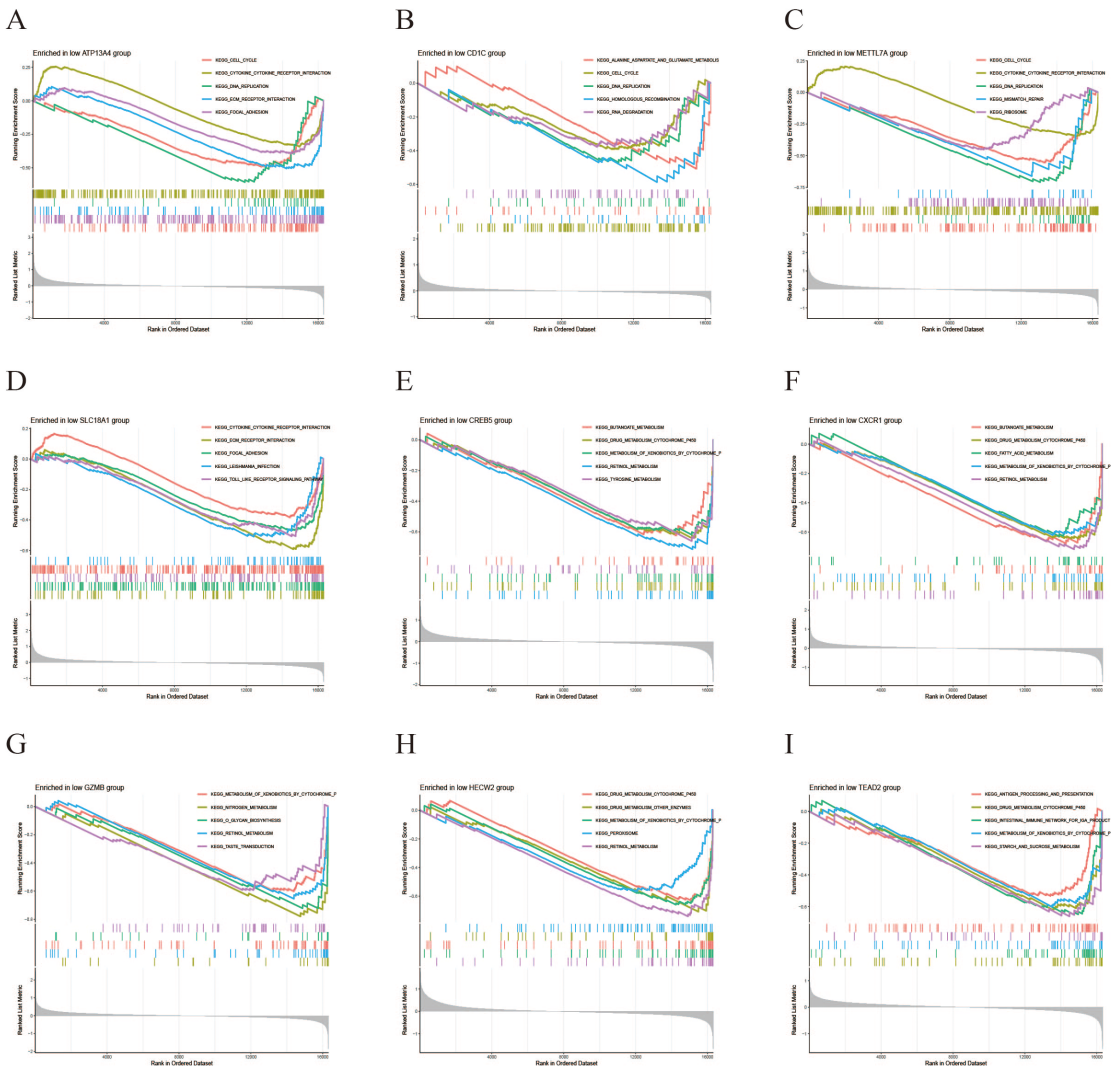
GSEA for low expression levels of disease-critical genes in CRC. (A–I) GSEA enrichment results for low expression of *ATP13A4, CD1C, METTL7A, SLC18A1, CREB5, CXCR1, GZMB, HECW2, TEAD2*.

GSEA was performed to investigate the functional pathway activities associated with high and low expression levels of the key genes in CRC. In the high-expression group, the “Cytokine-cytokine receptor interaction” pathway exhibited the highest enrichment, followed by “Toll-like receptor signaling”, “ECM receptor interaction”, and “Drug metabolism cytochrome P450” (Fig. 8). The low-expression group showed highest enrichment in the “Metabolism of xenobiotics by cytochrome” pathway, followed by “Drug metabolism cytochrome P450”, “Cytokine-cytokine receptor interaction”, and “Retinol metabolism” (Fig. 9). Notably, both expression groups exhibited enrichment in the “Cytokine-cytokine receptor interaction” and “Drug metabolism cytochrome P450” pathways for the majority of the key genes, suggesting their potential involvement in modulating intercellular interactions and metabolic processes in CRC.

### Immune cell infiltration analysis in CRC and immunological correlates of key genes

**Figure 10:**
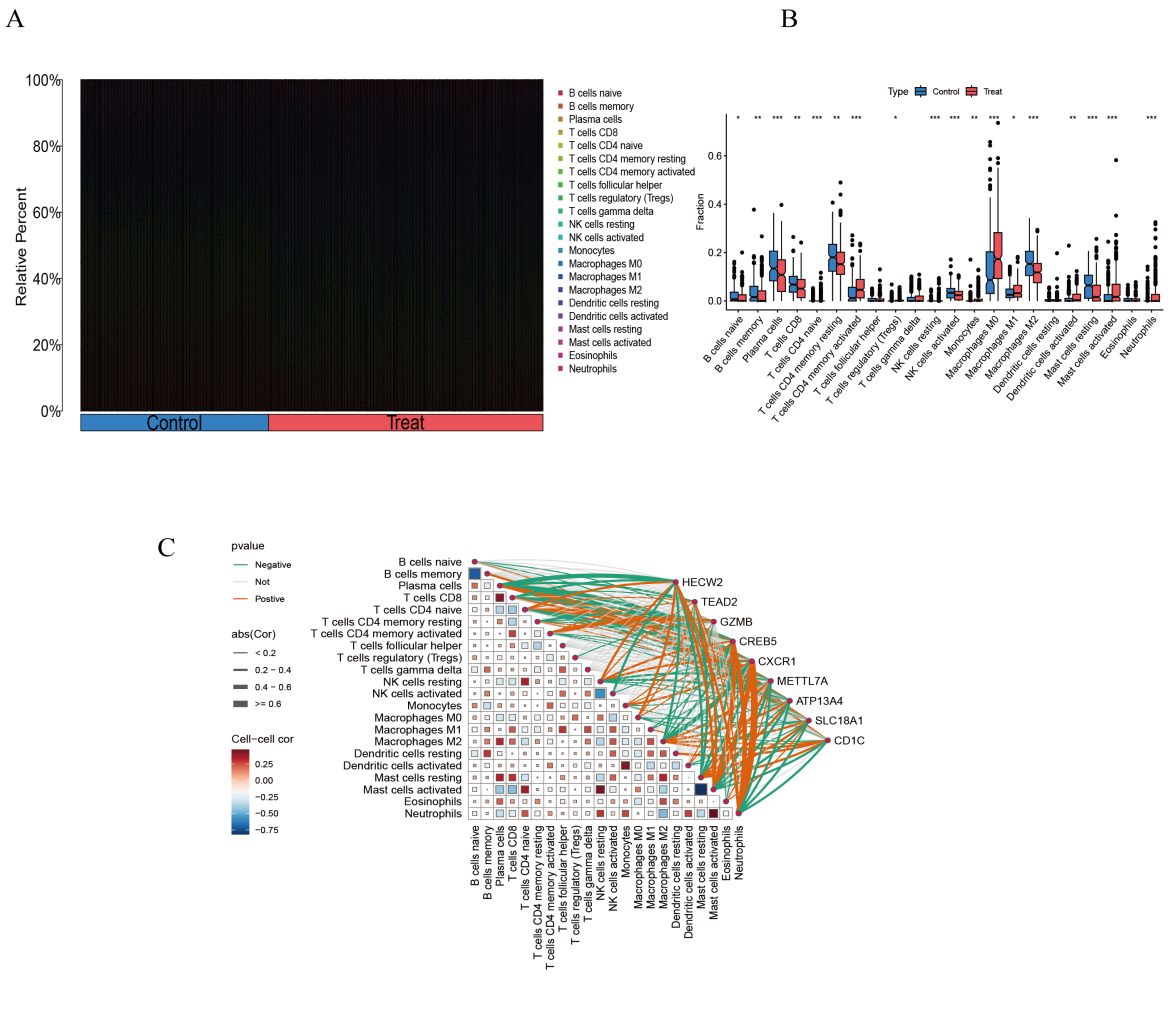
Immune infiltration analyses and correlations between disease–critical genes and infiltrating immune cell types. (A–B) Differential immune cell infiltration between control and colorectal cancer groups. (C) Correlation heatmap between disease–critical genes and infiltrated immune cells. *P < 0.05, **P < 0.01, ***P < 0.001.

Next, we applied the CIBERSORT algorithm to explore the immune cell infiltration differences between CRC and normal samples. CRC tissues exhibited significantly higher proportions of activated CD4 memory T cells, M0 macrophages, activated mast cells, and neutrophils. In contrast, significant reductions were observed in plasma cells, activated NK cells, M2 macrophages, and resting mast cells (Figs. 10A–10B). These results indicated that CD4+ T cells, macrophages, mast cells, and neutrophils might play important roles in CRC pathogenesis.

Correlation analysis between key gene expression and immune cell infiltration revealed significant positive correlations between activated mast cells and resting NK cells, activated mast cells and neutrophils, and activated dendritic cells and monocytes. Conversely, a significant negative correlation was observed between activated and resting mast cells. Furthermore, neutrophils and activated mast cells showed significant positive correlations with *HECW2*, *CREB5*, *CXCR1*, but significant negative correlations with *METTL7A*, *ATP13A4*, *SLC18A1*, and *CD1C*. Plasma cells exhibited significant negative correlations with *HECW2*, *TEAD2*, and *GZMB*, but a positive correlation with *CXCR1* (Fig. 10C).

These findings suggested significant associations between CRC key genes and specific immune cell populations within the tumor microenvironment. Notably, *CREB5* and *CXCR1* displayed widespread correlations with multiple immune cell types, highlighting their prominent roles in the immunological landscape of CRC.

### SMR analysis identifies candidate genes associated with CRC risk

**Figure 11:**
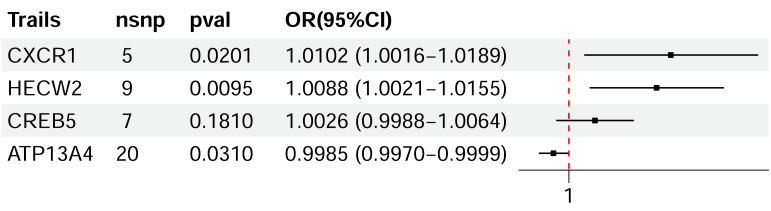
SMR results for candidate genes in CRC. Forest plot showing ORs and 95% CIs derived from SMR analysis. The dashed line indicates OR = 1.

SMR analysis was conducted to explore the potential causal associations between genetically predicted expression levels of candidate genes and colorectal cancer risk (Fig. 11). Increased genetically predicted expression of *CXCR1* was significantly associated with a higher risk of colorectal cancer (OR = 1.0102, 95% CI [1.0016–1.0189], *P* = 0.0201), and *HECW2* expression also exhibited a significant positive association with colorectal cancer susceptibility (OR = 1.0088, 95% CI [1.0021–1.0155], *P* = 0.0095). In contrast, no statistically significant association was observed between *CREB5* expression and colorectal cancer risk (OR = 1.0026, 95% CI [0.9988–1.0064], *P* = 0.1810). Notably, higher genetically predicted expression of *ATP13A4* was associated with a reduced risk of colorectal cancer (OR = 0.9985, 95% CI [0.9970–0.9999], *P* = 0.0310). Overall, these results indicate that *CXCR1* and *HECW2* may act as risk-associated genes, whereas *ATP13A4* may exert a protective role in colorectal cancer, while CREB5 did not show evidence of a significant association.

### Validation of CRC key genes in cell lines

**Figure 12:**
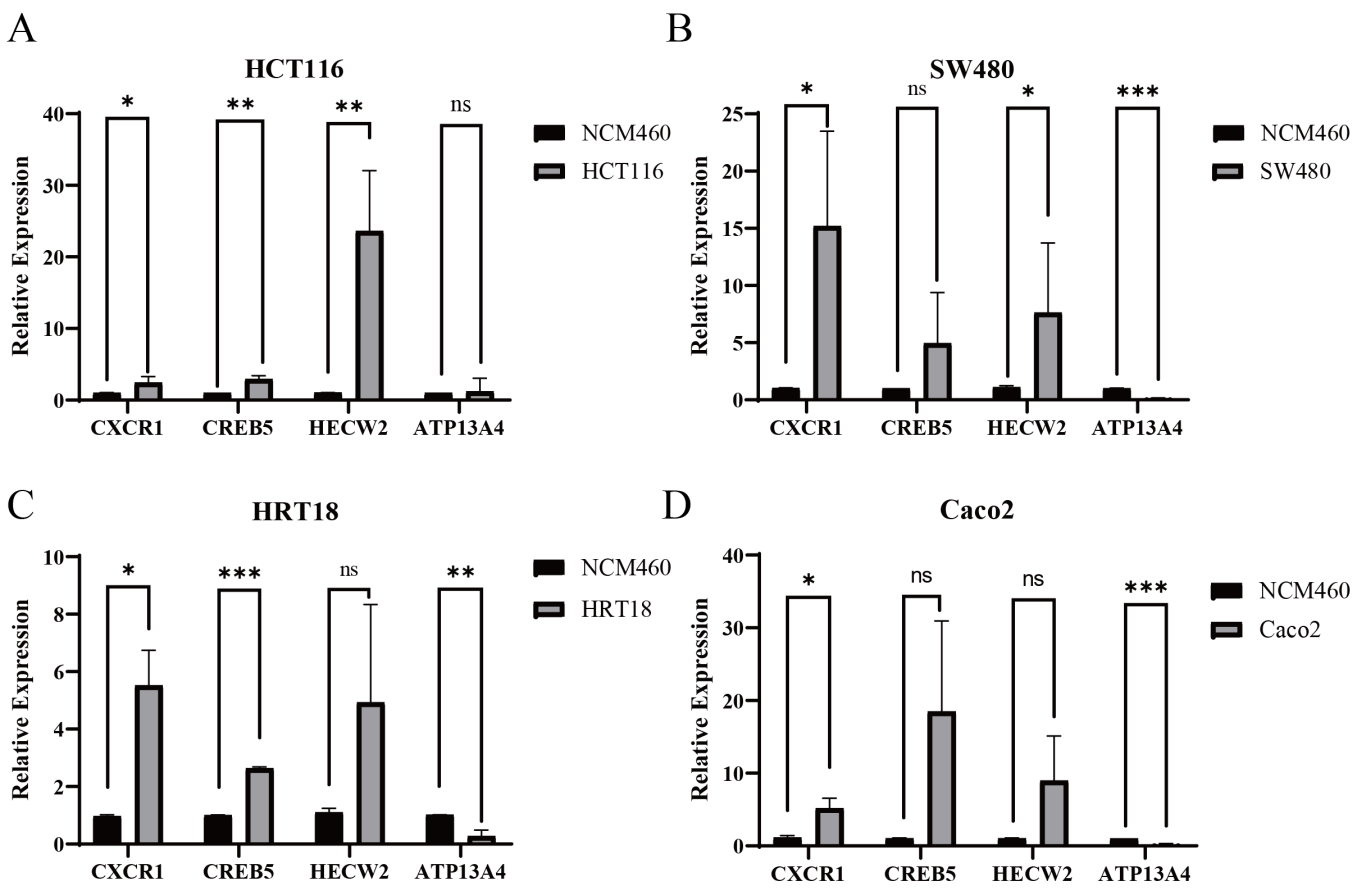
Relative expression levels of key genes in CRC cell lines. Quantitative reverse transcription polymerase chain reaction (qRT-PCR) was used to detect the relative mRNA expression levels of CXCR1, CREB5, HECW2, and ATP13A4 in CRC cell lines HCT116 (A) SW480 (B) HRT18 (C) and Caco2 (D) with comparison to the normal colorectal epithelial cell line NCM460. Data are presented as mean ± SD. Statistical analysis was performed using Student’s *t*-test; * *P* < 0.05, ** *P* < 0.01, *** *P* < 0.001; ns, no statistically significant difference.

To further validate the candidate genes prioritized by SMR analysis at the cellular level, quantitative reverse transcription polymerase chain reaction (qRT-PCR) was performed to examine the mRNA expression levels of *CXCR1*, *CREB5*, *HECW2*, and *ATP13A4* in colorectal cancer cell lines HCT116, SW480, HRT18, and Caco2, with the normal colorectal epithelial cell line NCM460 used as a control. The results showed that in HCT116 cells, the expression levels of *CXCR1*, *CREB5*, and *HECW2* were significantly higher than those in NCM460 cells, whereas no significant difference was observed for ATP13A4 (Fig. 12A). In SW480 cells, *CXCR1* and *HECW2* were significantly upregulated, while *ATP13A4* was significantly downregulated, and *CREB5* showed no significant difference compared with NCM460 (Fig. 12B). In HRT18 cells, *CXCR1* and *CREB5* expression levels were significantly increased, whereas *ATP13A4* was significantly downregulated, and *HECW2* expression did not differ significantly (Fig. 12C). In Caco2 cells, *CXCR1* expression was significantly upregulated and *ATP13A4* was significantly downregulated, while no significant differences were observed for *CREB5* or *HECW2* (Fig. 12D). Collectively, these qRT-PCR results demonstrate that the expression patterns of the SMR-identified candidate genes vary across different CRC cell lines, indicating a degree of cell line–specific heterogeneity, and provide experimental support for the findings of the SMR analysis.

## Discussion

CRC remains one of the most prevalent and lethal malignancies worldwide, and its global burden continues to increase despite advances in early screening and multimodal treatment strategies. The rising incidence of CRC, together with its marked molecular and immunological heterogeneity, highlights the limitations of current clinicopathological indicators in accurately predicting disease progression and therapeutic response. Consequently, the identification of robust molecular biomarkers that integrate genetic susceptibility, transcriptional dysregulation, and immune contexture has become increasingly important for improving precision diagnosis and prognostic stratification. In this study, we addressed this unmet need by applying an integrative analytical framework combining transcriptomic profiling, Mendelian randomization, and immune infiltration analysis to systematically identify CRC-associated key genes. Our findings provide a comprehensive genetic and immunological perspective on CRC pathogenesis and establish a foundation for exploring novel biomarkers and therapeutic targets.

CRC is a highly heterogeneous disease. Emerging evidence suggests that immune checkpoints have potential research and clinical relevance in CRC, as high microsatellite instability (MSI) is commonly associated with increased tumor mutational burden and enhanced immune cell infiltration, and has been linked to responses to immunotherapy (Ganesh et al., 2019). In this research, we identified distinct immune cell infiltration patterns between normal and CRC tissues, characterized by a significant decrease of plasma cells and an increase of activated CD4+ T cells in CRC. Plasma cells, specialized for synthesizing and secreting large quantities of antigen-specific immunoglobulins (Igs), are pivotal mediators of humoral immunity (Roth et al., 2014; Tangye et al., 2023). Conversely, accumulating evidence indicates that naive CD4+ T lymphocytes can spontaneously acquire a memory phenotype and exhibit innate immune functions independently of antigen recognition, thereby augmenting host defense against pathogens (Kawabe & Sher, 2022). This observed reduction in plasma cells coupled with an elevation in CD4+ T lymphocytes suggests a potential suppression of humoral immunity concurrent with activation of cellular immunity in CRC. The attenuation of humoral responses may contribute to CRC pathogenesis, while the significant increase in activated CD4+ T memory cells likely plays a promotive role, potentially linked to improved prognosis, consistent with previous findings (Fidelle et al., 2020).

To further strengthen the causal relevance of our findings, SMR analysis was performed, which prioritized four CRC-associated genes (*CXCR1, CREB5, HECW2,* and *ATP13A4*). Subsequent qRT-PCR validation in multiple CRC cell lines (HCT116, HRT18, SW480, and Caco-2) confirmed the consistent dysregulation of these genes, supporting the reliability of the integrative analytical framework and highlighting their potential roles in CRC progression. *CREB5* is a member of the *CREB* family, encoding transcription activators in eukaryotic cells (Hwang et al., 2022). Previous studies have shown that *CREB5* expression is upregulated in CRC tissues and cell lines, and *CREB5* can directly activate Mesenchymal-Epithelial Transition Factor (MET) to drive CRC invasion and metastasis (Li et al., 2020; Wang et al., 2020). *CXCR1*, a key member of the G-protein-coupled receptor (GPRC) family, serves as one of the main receptors for the CXC chemokine interleukin-8 (IL-8) (Liu et al., 2016). A study conducted by Łukaszewicz-Zając et al. (2023) has indicated that *CXCR1* and *CXCR2* may represent potential therapeutic targets for CRC. Furthermore, *CXCL1* and *CXCL8*, *via* their cognate receptors, mediate tumor growth, angiogenesis, and metastasis in CRC and other malignancies (Verbeke et al., 2011). Notably, small molecule antagonists targeting CXCR1/CXCR2 can inhibit CRC liver metastasis (Varney et al., 2011). *HECW2*, a member of the E3 ubiquitin ligase family, is highly expressed in CRC tissues. *HECW2* can activate the AKT/mTOR signaling pathway by mediating the ubiquitin-proteasome degradation of lamin B1, thereby promoting CRC progression and chemoresistance (Li et al., 2023).

At present, evidence regarding the expression pattern and functional role of *ATP13A4* in CRC remains extremely limited, representing a clear knowledge gap in the current literature. Given that *ATP13A4* is a P5B-type ATPase and a paralog of *ATP13A2*—whose oncogenic role in CRC has been established through activation of the pentose phosphate pathway (PPP)—it is reasonable to hypothesize that *ATP13A4* may exert related or complementary functions in CRC progression (Chen et al., 2021; Gibbons et al., 2020). Emerging studies in breast cancer have demonstrated that *ATP13A4* upregulation enhances the polyamine transport system, thereby supporting cancer cell proliferation (Van Veen et al., 2023). As CRC development is highly dependent on metabolic reprogramming, including dysregulated polyamine metabolism and PPP activation, *ATP13A4* may contribute to tumor growth by modulating intracellular polyamine homeostasis and associated metabolic pathways. In addition, ATP13A4-mediated activation of the JNK signaling pathway, which is known to participate in CRC progression, suggests an alternative mechanism through which *ATP13A4* may influence tumor behavior (Van Veen et al., 2023). Although direct experimental evidence is currently lacking, these observations collectively support the notion that *ATP13A4* may represent a previously unrecognized regulator of CRC progression and a potential diagnostic biomarker or therapeutic target. Further mechanistic and clinical studies are warranted to validate this hypothesis.

In summary, both high-risk and low-risk genes play crucial roles in CRC pathogenesis. Further investigation of high-risk genes may facilitate the development of novel inhibitors and targeted therapies. For low-risk genes, identifying activators to enhance their tumor-suppressive functions is equally important. Notably, the genes we identified in this study are intricately linked to tumor immunity, suggesting their potential as targets for immunotherapeutic strategies. This study provides important insights into the genetic mechanisms underlying CRC and identifies a set of immune-related genes that may be involved in regulating tumor progression and therapeutic response. Although immune checkpoint inhibitors (ICIs) have shown promising prospects in CRC treatment, their efficacy prediction requires a multi-biomarker panel to improve accuracy. The core genes identified in this study, particularly those involved in immune regulation, could serve as potential candidates for inclusion in such panels. Additionally, the study observed changes in immune cell infiltration patterns in CRC tissues, including a reduction in plasma cells and an increase in activated CD4+ memory T cells. This shift suggests that cellular immunity plays an important role in CRC development and may provide valuable insights for the clinical application of immune checkpoint inhibitors.

Nevertheless, several limitations of this study should be objectively acknowledged. First, the relatively small sample size of the eQTL datasets may have reduced the statistical power of the MR analyses. Second, the GWAS data were mainly derived from populations of European ancestry, which might limit the generalizability of the findings to other ethnic groups. In addition, although FDR correction was applied to control for multiple testing in the MR analyses, some candidate gene associations did not reach the conventional threshold for statistical significance after adjustment and therefore should be interpreted with caution.

Notably, the MR associations for *CREB5* and *HECW2* remained statistically significant after FDR correction, supporting the robustness of the main findings, whereas the associations for *ATP13A4* and *CXCR1* did not retain significance after adjustment but showed consistent effect directions, suggesting borderline or suggestive evidence that warrants further validation. *CREB5* showed a *P* value of 0.1810 in the SMR analysis, which did not reach statistical significance. This suggests that a causal association between *CREB5* and CRC was not evident in the dataset analyzed. Nevertheless, considering the reported potential roles of *CREB5* in tumor-related signaling pathways, it may still hold research interest in CRC pathogenesis and warrants further functional investigation. Consistently, at the experimental validation level, several core genes did not exhibit statistically significant expression differences in specific cell lines (*P* > 0.05), including *ATP13A4* in HCT116 cells, *HECW2* in HRT18 cells, *CREB5* in SW480 cells, and both *CREB5* and *HECW2* in Caco-2 cells. Despite the lack of statistical significance, the observed expression trends were largely consistent with the bioinformatics and MR results, indicating potential biological relevance. These discrepancies may reflect intrinsic biological heterogeneity among different cell lines as well as limited sample size, and further validation in larger population cohorts and more diverse experimental models is warranted.

To address these limitations, future research should focus on the following: Firstly, expanding the sample size will enhance dataset accuracy, reliability and result generalizability. Secondly, validating genes that lacked significance in initial cellular experiments requires assessment across multiple cell lines, complemented by multi-omics analyses to elucidate their expression patterns and regulatory mechanisms in diverse contexts. Finally, further optimization of the predictive model is needed to identify key genes with the highest predictive value, and subsequent functional validation through comprehensive *in vitro* and *in vivo* experiments will enhance the accuracy and robustness of the model.

## Conclusion

Comprehensive analyses indicate that the high-risk genes *CXCR1* and *HECW2*, as well as the low-risk gene *ATP13A4,* may be biologically relevant to CRC development and progression. Our findings provide a foundation for developing novel targeted and immunotherapeutic strategies. However, limitations include the sample size and the lack of statistical significance in certain cellular experiments. Future research should focus on expanding the sample size, validating these findings in additional cellular models, and incorporating multi-omics analyses to fully elucidate the molecular mechanisms and clinical relevance of these key genes in CRC.

## Supplemental Information

10.7717/peerj.21070/supp-1 Supplemental Information 1 Leave-one-out analysis of the association between disease critical genes and colorectal cancer (A-I) *ATP13A4, CD1C, METTL7A, SLC18A1, CREB5, CXCR1, GZMB, HECW2, TEAD2.*

10.7717/peerj.21070/supp-2 Supplemental Information 2 GSE110224 dataset as well as the gene expression dataset of CRC in the TCGA database for external test of the association between disease critical genes and colorectal cancer (A-I) *ATP13A4, CD1C, METTL7A, SLC18A1, CREB5, CXCR1, GZMB, HECW2, TEAD2.*

10.7717/peerj.21070/supp-3 Supplemental Information 3 Supplemental tables

10.7717/peerj.21070/supp-4 Supplemental Information 4 Raw data GSE9348

10.7717/peerj.21070/supp-5 Supplemental Information 5 Raw data GSE87211

10.7717/peerj.21070/supp-6 Supplemental Information 6 Raw data GSE83889

10.7717/peerj.21070/supp-7 Supplemental Information 7 Raw data of GSE110224

10.7717/peerj.21070/supp-8 Supplemental Information 8 Raw data of differential expression gene analysis

10.7717/peerj.21070/supp-9 Supplemental Information 9 Raw data of GO enrichment analysis

10.7717/peerj.21070/supp-10 Supplemental Information 10 Raw data of KEGG enrichment analysis

10.7717/peerj.21070/supp-11 Supplemental Information 11 CIBERSORT-Results

10.7717/peerj.21070/supp-12 Supplemental Information 12 MIQE checklist

## Additional Information and Declarations

### Data Availability

The following information was supplied regarding data availability: The raw measurements are available in the Supplemental Files. The data are available in the NCBI Gene Expression Omnibus (GEO): GSE9348, GSE83889, GSE87211, and GSE110224. The mRNA expression data for colorectal cancer (CRC) were obtained from The Cancer Genome Atlas (TCGA) database as an external independent validation cohort. The data were retrieved *via* the Genomic Data Commons (GDC) Data Portal using the following search criteria: - Project: TCGA-COAD and TCGA-READ
- Data Category: Transcriptome Profiling - Data Type: Gene Expression Quantification - Workflow Type: HTSeq-FPKM
