# Supplementary figures and images for: Identification of colorectal cancer immune biomarkers via eQTL mapping: a Mendelian randomization and transcriptomic analysis study
Source: PeerJ. 2026 Apr 14;14:e21070. doi: 10.7717/peerj.21070 (PMC13089222; doi:10.7717/peerj.21070)

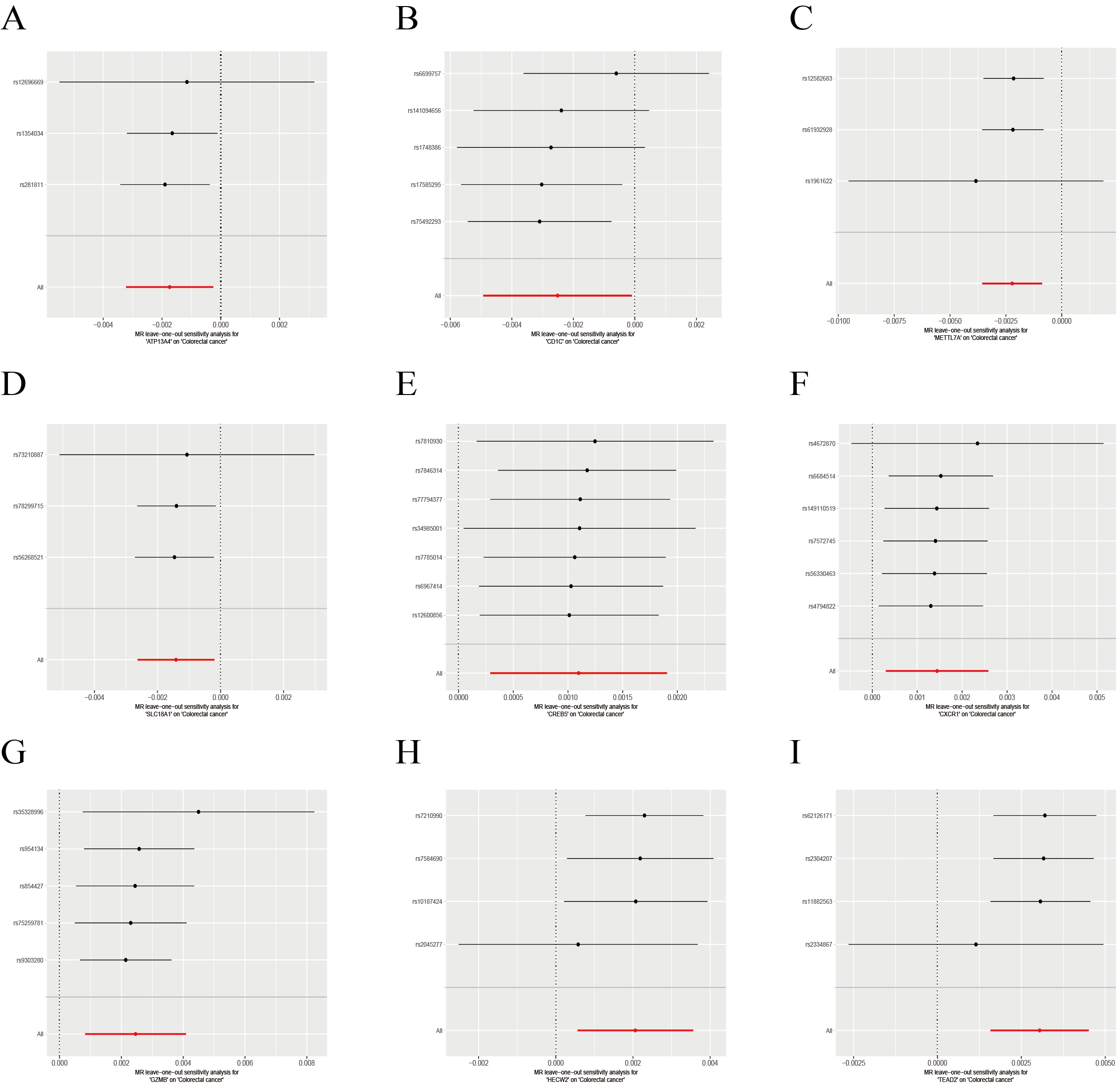

Supplement: Supplemental Information 1 — (A-I) ATP13A4, CD1C, METTL7A, SLC18A1, CREB5, CXCR1, GZMB, HECW2, TEAD2. [file peerj-14-21070-s001.png]

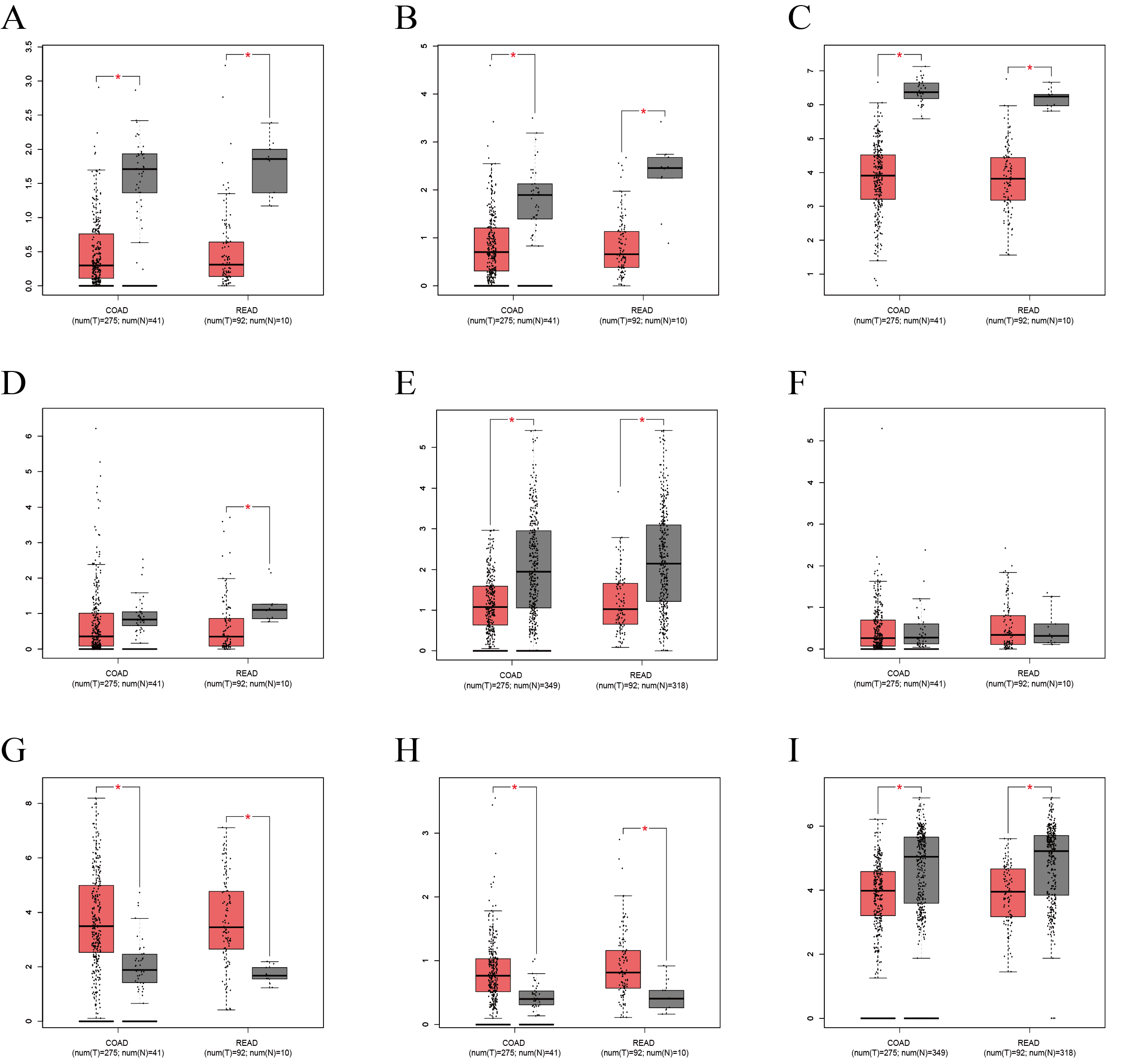

Supplement: Supplemental Information 2 — (A-I) ATP13A4, CD1C, METTL7A, SLC18A1, CREB5, CXCR1, GZMB, HECW2, TEAD2. [file peerj-14-21070-s002.png]
